# How personality influences health outcomes and quality of life in adult patients with cystic fibrosis

**DOI:** 10.1186/s12890-023-02463-y

**Published:** 2023-06-01

**Authors:** Ute Niehammer, Svenja Straßburg, Sivagurunathan Sutharsan, Christian Taube, Matthias Welsner, Florian Stehling, Raphael Hirtz

**Affiliations:** 1 Department of Pulmonary Medicine, Adult Cystic Fibrosis Center, University Hospital Essen - Ruhrlandklinik, University of Duisburg-Essen, Essen, Germany; 2 Devision of Pediatric Pulmonology and Sleep Medicine, Department of Pediatrics III, University Hospital Essen, University of Duisburg-Essen, Essen, Germany; 3 Department of Pediatrics, Division of Rare Diseases and CeSER, St. Josef-Hospital, Ruhr-University Bochum, Bochum, Germany; 4 Division of Pediatric Endocrinology and Diabetology, Department of Pediatrics II, University Hospital Essen, University of Duisburg-Essen, Essen, Germany

**Keywords:** Cystic fibrosis, Personality traits, Health-related quality of life, Clinical outcomes

## Abstract

**Background:**

The present study evaluates personality traits in adult patients with cystic fibrosis (CF) and correlates these results with health-related quality of life (HRQoL) and other clinical parameters indicative of disease severity.

**Methods:**

Seventy adults completed the Cystic Fibrosis Questionnaire-Revised (CFQ-R 14+), a CF-specific measure of HRQoL, and a self-administered questionnaire about personality traits and disorders. Mean subscale scores and the prevalence of extreme personality traits on the `Persönlichkeits-Stil- und Störungs-Inventar (PSSI)´ were compared to the norming sample. Moreover, a cluster analysis was conducted to identify personality styles among people with cystic fibrosis (pwCF). The relationship between mean PSSI subscale scores and personality clusters with HRQoL and clinical outcomes, e.g., percent predicted forced expiratory volume in one second (ppFEV_1_), and body mass index (BMI), was studied by regression analysis considering important confounders.

**Results:**

On several of the subscales of the personality questionnaire, people with cystic fibrosis (pwCF) showed either significantly higher or lower scores than the norm sample. In further analyses, two personality clusters could be identified. PwCF from the cluster with predominantly low scores on the subscales ‘negativistic’, ‘schizoid’, ‘borderline’, ‘depressed’, and ‘paranoid’ showed better HRQoL than pwCF from the other cluster with mainly high normal or elevated scores. The studied health outcomes proved to be independent of the respective personality clusters.

**Conclusions:**

In pwCF, HRQoL is mainly determined by psychological factors, including personality. Since more recent personality theories assume that personality is modifiable, our findings imply that patients with accentuated personality traits may benefit from psychosocial support.

**Supplementary Information:**

The online version contains supplementary material available at 10.1186/s12890-023-02463-y.

## Introduction

Cystic fibrosis (CF) is the most common rare inherited disease in the Caucasian population. CF is a life-limiting and incurable metabolic disease caused by a gene mutation leading to dysfunction of the ‘cystic fibrosis transmembrane conductance regulator (CFTR) protein’. This affects multiple organ systems, particularly the lungs, pancreas, upper respiratory tract, liver, intestines, and reproductive organs.

With the increasing life expectancy of people with CF (pwCF) [1], psychosocial aspects of the disease have gained greater importance due to a significant disease- and treatment-related burden [2]. Thus, numerous studies have addressed health-related quality of life (HRQoL) in pwCF, a multidimensional concept that assesses health status by recording physical health, psychological well-being, and social integration from the patient's perspective. While medical parameters, such as percent predicted forced expiratory volume in one second (ppFEV_1_) are only moderately associated with HRQoL [3], mental health variables such as depression and anxiety play a more critical role in predicting HRQoL [4]. Recent work also demonstrates the importance of previously understudied behaviors in pwCF, such as cough suppression (CS) – a behavior characterized by suppressing coughs in public to avoid negative attention despite the urge to cough [5].

Studies in chronically ill patients have shown that HRQoL is also influenced by specific personality traits [6]. For example, patients with high loadings on ‘neuroticism’ show an exceptionally high disease burden [7]. Furthermore, personality traits are shown to be stronger determinants of HRQoL than sociodemographic or clinical variables [8].

However, personality traits may also affect clinical parameters, including ppFEV_1_, in chronic lung disease [9]. Nonetheless, only a few studies exist concerning personality traits in pwCF and their psychological and outcome-related medical impact [10]. Moreover, previous studies employed tests not intended to assess personality in the normative range but to focus on psychopathological aspects of personality. Thus, these tests were likely insufficiently discriminating to comprehensively assess the personality of pwCF.

The present study aims to describe the relationship between personality and health outcomes (ppFEV_1_ and BMI) and HRQoL in adult pwCF. We hypothesize that, in line with adult patients with other chronic diseases [11–13], pwCF show a higher prevalence of more extreme personality traits that affect their physical and psychological level of functioning. Moreover, beyond a comparison of pwCF to the general population, the present study is also intended to identify certain personality clusters among pwCF. Knowledge about personality traits in pwCF could help to individualize and thereby increase the effectiveness of supportive interventions. This could result in improved HRQoL and health outcomes.

## Method

### Study design

In this cross-sectional study, patients of the ‘Adult Cystic Fibrosis Center, University Hospital Essen—Ruhrlandklinik, Germany’ were approached to participate during an outpatient or inpatient stay between January 2019 and March 2020. Ethical approval was granted by the Ethics Committee of the University Duisburg-Essen (18–8412-BO). All subjects provided informed written consent to the study, answered a personality and HRQoL questionnaire, and provided personal demographic information. Clinical data were collected by the attending physicians during the visit/stay.

### Subjects

Patients who had undergone lung transplantation and patients who could not complete the questionnaires without assistance were excluded. Except for three pwCF who denied participation, the study included all outpatients and inpatients over 18 years of age with a confirmed diagnosis of CF attending the ‘Adult Cystic Fibrosis Center, University Hospital Essen – Ruhrlandklinik’ between January 2019 to March 2020 [14].

### Measures—*disease status*

The attending physician recorded disease status, including ppFEV_1_, BMI, genotype, Pseudomonas aeruginosa colonization status, pancreatic function status, and the presence of CF-related diabetes mellitus.

### Measures—*personality*

Personality was assessed by the revised ‘Persönlichkeits-Stil- und Störungs-Inventar’ (PSSI), a well validated self-report questionnaire for adolescents 14 years and older and adults [15]. The questionnaire contains 140 items related to 14 scales (see Additional file 1—Table 1). Answers are provided on a four-point Likert scale within the poles ‘strongly disagree’ to ‘strongly agree’. The questionnaire conceptualizes personality traits as non-pathological equivalents to personality disorders from DSM-IV and ICD-10. For any clinical disorder category, there is an analogous personality style, which can be described by interindividual differences on a corresponding quantitatively graded dimension. However, a personality trait recorded in its extreme expression represents a potential risk for a specific personality disorder. The questionnaire shows good psychometric properties. Internal consistency is given (above 0.8) and construct validity is acceptable for clinical and non-clinical behavior [15]. The norms are based on the general German population, including 1903 adults aged 18 to 82 and 40 adolescents aged 14 to 17.

### Measures—*health-related quality of life*

The revised ‘Cystic Fibrosis Questionnaire for adolescents and adults over 14 years old (CFQ-R 14 +)’ was used to assess HRQoL. This CF-specific, self-report questionnaire consists of 50 items on 12 scales and includes generic and disease-specific dimensions as well as a scale for the evaluation of subjective health. Each domain is standardized on a 0–100 scale with higher scores indicating better HRQoL. The German version of the CFQ-R 14 + shows good psychometric properties [16].

### Measures—*questionnaire on CS, therapy adherence, and demographic variables*

To gain information about cough suppression (CS), therapy adherence, and demographic variables, participants completed a self-report questionnaire developed by the research team [17]. The survey includes 12 items that measure the participants’ assessment of future health perspectives (one item), cough frequency, and sputum quantity (two items), CS (one item), therapy adherence (three items), and diagnosis disclosure (four items, Additional file 1—Table 2). In addition, the participants were asked to specify their educational, employment, and marital status.

### Statistical analyses

Data handling and statistical analyses were performed with SPSS 27.0 (IBM Corp., Armonk, NY). All analyses were corrected for multiple testing controlling the two-tailed false-discovery-rate (FDR) at q < 0.05 [18], except for exploratory analyses comparing PSSI subscale means between personality clusters and those analyses investigating the relationship between personality clusters and CFQ-R subscale scores. Effect size calculations (d, semipartial correlation (sr^2^), partial η^2^ (pη^2^), odds ratio (OR)) relied on SPSS. For ease of interpretation, effect size measures were converted to Cohen’s d (small 0.2 ≤ d < 0.5, medium 0.5 ≤ d < 0.8, large d ≥ 0.8) using an online calculator (d) [19].

Details on testing the assumptions of all outlined statistical procedures are provided in the Additional Material.

#### Single scale analysis

The central tendency of each PSSI subscale was tested against a mean of 50 by one-sample t-tests as the PSSI subscale scores follow a t-distribution with a mean of 50 and a standard deviation (SD) of 10. In the presence of outliers (values exceeding ± 2.5 the median absolute difference [20]), one-sample Wilcoxon tests were performed to compare subscale scores to a median of 50.

We used detailed information regarding the distribution of PSSI subscale t-scores from the manual to separately compare the frequency of scores below and above the normal range of PSSI scores (40 – 60) between the norming sample and the sample of pwCF by χ^2^-tests of independence. All analyses were checked for sufficient cell size.

The linear relationship between ppFEV_1_, BMI, and CFQ-R overall score as well as therapy adherence (dependent variables) with the PSSI subscale t-scores (independent variables) was assessed by multiple regression considering an analysis-specific subset of covariates with significant correlations regarding the dependent variable of interest (please see the Additional Material). Considering the scale of measure of CS (dependent variable), its relationship with the PSSI subscales (independent variables) was determined by ordinal logistic regression relying on a logit function, likewise accounting for important covariates. Please note, that prior to analysis, all categorical variables were dummy coded (please see the Additional Material).

#### Cluster analysis

To identify certain personality styles *among* pwCF irrespective of differences compared to the norming sample, the PSSI subscale t-scores were subjected to a two-step cluster analysis using a log-likelihood distance measure for clustering and the Akaike Information Criterion (AIC) to determine the maximum number of clusters with a preset of no more than 10 clusters.

The resulting clusters (independent variable) were compared regarding ppFEV_1_, BMI, CFQ-R overall score, and therapy adherence (dependent variables) by an analysis of covariance (ANCOVA) considering the same analysis-specific subset of covariates as detailed above. The relationship between personality clusters and CS was evaluated by logistic regression.

## Results

Of the 72 subjects with CF participating in the present study, 70 were considered for analysis as two cases did not complete the PSSI. Regarding the CFQ-R results, data from 63 participants were available for analysis. Participants who did not fully complete the CFQ-R did not differ from participants with complete data regarding demographic and clinical variables (s. Table 1 and Additional file 1—Table 3).

**Table 1:** Patient demographic and clinical characteristics, *n* = 70

	All subjects (*n* = 70)
Age, years	32.71 ± 11.65 (18–71)
Sex	41 (59) male
Genotype n, (%)	
*F508del homozygous*	28 (40)
*F508del heterozygous*	31 (44)
*Other*	11 (16)
BMI, kg/m^2^	20.32 ± 3.41 (15–33)
ppFEV_1_	43.27 ± 19.93 (16–99)
Pancreatic insufficiency n, %	45 (64)
CF-related diabetes n, %	22 (31)
P. aeruginosa positive n, %	52 (74)
Hospital n, %	
*Inpatient*	67 (96)
*Outpatient*	3 (4)
Reasons for medical treatment n, %	
*PEX*	33 (47)
*Starting CFTR-Modulator Therapy*	14 (20)
*IVAT*	8 (11)
*Others*	16 (22)
Marital status n,%	
*Single*	36 (51)
*In partnership / married*	34 (49)

The results are presented as the mean ± standard deviation (SD) and range or number of pwCF n (%)

*BMI* Body mass index, *ppFEV1* percent predicted forced expiratory volume in one second, *PEX* Pulmonary exacerbation, *IVAT* Intravenous antibiotic therapy (prophylactic)

### Demographics and clinical data

The subjects' demographic and clinical data are summarized in Table 1. The mean age was 32.7 years (SD 11.65, range 18–71), and 59% of the sample was male. Most of the subjects were hospitalized (96%). The average BMI was 20.32 kg/m^2^ (SD 3.41). Lung function indicated moderate disease severity (average ppFEV_1_: 43.27, SD 19.93). Almost half (47%) received hospital treatment because of pulmonary exacerbation, 20% started CFTR-modulator therapy, 11% took prophylactic antibiotic therapy, and 22% were hospitalized for various causes.

### Single scale analysis – comparison to the norming sample of PSSI

**Fig.1:**
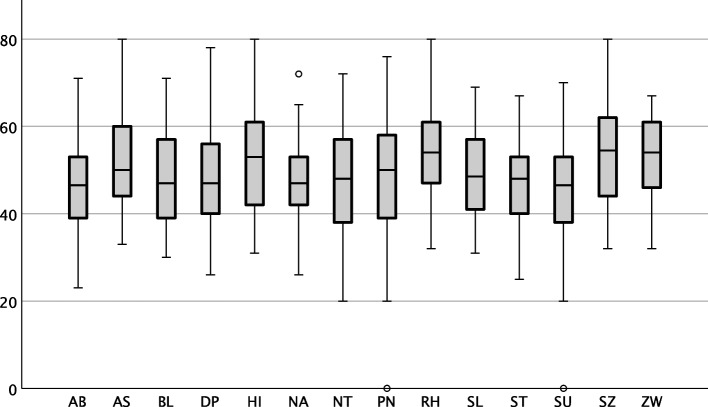
Boxplots of PSSI subscale scores in pwCF. The horizontal line indicates the scale mean. *PN* Paranoid, *SZ* Schizoid, *ST* Schizotypal, *BL* Borderline, *HI* histrionic, *NA* Narcissistic, *SU* Self-insecure, *AB* Dependent, *ZW* Compulsive, *NT* Negativistic, *DP* Depressed, *SL* Selfless, *RH* Rhapsodic, *AS* Antisocial. Note, that there were outliers for the PN, NA, and SU subscales

Considering a correction for multiple comparisons, pwCF were found to have higher t-scores on the subscale 'rhapsodic (RH)' (exaggerated form of a positive attitude of life that goes along with the inability handling conflicts and problems; M = 54.5, *p* = 7 × 10^–4^, d = 0.43; Table 2, Fig. 1) and lower t-scores on the subscale 'self-insecure (SU) (fear of negative evaluation by peers, shyness, and social insecurity; M = 45.1, *p* = 0.002, d = 0.37) compared to a scale mean of 50. Moreover, there was a trend for a significant difference between the PSSI t-scores of pwCF and a scale mean of 50 regarding the subscales ‘schizoid (SZ)’ (M = 53.5, *p* = 0.02, d = 0.29), ‘schizotypal (ST)’ (M = 47.4, *p* = 0.02, d = 0.29), ‘narcissistic (NA)’(M = 47.4, *p* = 0.02, d = 0.29), ‘dependent (AB)’ (M = 46.7, *p* = 0.007, d = 0.33), and ‘compulsive (ZW)’ (M = 53.1, *p* = 0.008, d = 0.33).

**Table 2:** PSSI results. Descriptive results concerning the PSSI subscales in the total sample of pwCF, the two personality clusters among pwCF, and in the norming sample, including information on significant differences between the total sample of pwCF with the norming sample

	**pwCF** **(*N* = 70)**			**pwCF personality** **cluster 1** **(*N* = 41)**			**pwCF personality** **cluster 2** **(*N* = 29)**			**Norming sample** **(*N* = 1.943)**					
Scale	**Descriptives**	**Below the norm (%)**	**Above the norm (%)**	**descriptives**	**Below the norm (%)**	**Above the norm (%)**	**Descriptives**	**Below the norm (%)**	**Above the norm (%)**	**Descriptives**	**Below the norm (%)**	**Above the norm (%)**	***p*-value central tendency**	***p*-value % above**	***p*-value % below**
**PN**	47.97/50^a^ (13.72) [0—76]	25.7	15.7	54.90 (10.29) [20—76]	4.9	26.8	38.17 (11.99) [0—60]	55.2	0	50 (10) [23—77]	11.4	12.5	0.33	**0.001**^c^	0.46
**SZ**	53.46 (11.76) [32–80]	15.7	38.6	60.22 (8.80) [36—80]	4.9	61	43.90 (8.28) [32—66]	31	6.9	50 (10) [25—77]	11.5	12.7	0.02	0.26	**1 × 10**^**−7**d^
**ST**	47.43 (8.85) [25—67]	20	7.1	49.71 (8.83) [35—67]	12.2	12.2	44.21 (7.94) [25—60]	31	0	50 (10) [23—77]	11.8	13.1	0.02	0.06	0.2
**BL**	47.70 (10.54) [30–71]	30	11.4	53.73 (8.44) [39—71]	4.9	19.5	39.17 (6.60) [30—55]	65.5	0	50 (10) [26—77]	13.1	13.2	0.07	**2 × 10**^**−4**c^	0.86
**HI**	52.56 (11.51) [31—80]	17.1	25.7	49.44 (10.23) [31—69]	22	12.2	56.97 (11.95) [38—80]	10.3	44.8	50 (10) [23—77]	12.5	12.4	0.07	0.27	**0.003**^d^
**NA**	47.44/47^a^ (9.30) [26—72]	21.4	7.1	47.20 (8.51) [30—72]	17.1	4.9	47.79 (10.46) [26—72]	27.6	10.3	50 (10) [23—77]	11.3	12.7	0.02	0.02	0.2
**SU**	45.06/46.5^a^ (12.21) [0—70]	27.1	5.7	46.93 (13.09) [0—79]	24.4	9.8	42.41 (10.49) [20—58]	31	0	50 (10)	11.4	13.4	**0.002**^b^	**4 × 10**^**−4**c^	0.07
**AB**	46.73 (9.84) [23—71]	25.7	8.6	47.56 (10.20) [23—65]	26.8	9.8	45.55 (9.34) [30—71]	24.1	6.9	50 (10) [23—75]	12.8	13	0.007	**0.004**	0.36
**ZW**	53.09 (9.37) [32—67]	10	32.9	53.02 (10.16) [34—67]	14.6	36.6	53.17 (8.31) [32—65]	3.4	27.6	50 (10) [23—75]	13.7	13.1	0.008	0.48	**3 × 10**^**−5**d^
**NT**	47.89 (11.62) [20—72]	31.4	15.7	55.73 (7.43) [41—72]	0	26.8	36.79 (6.00) [20—49]	75.9	0	50 (10) [23—77]	11.9	13.1	0.13	**2 × 10**^**−5**c^	0.47
**DP**	47.91 (11.31) [26—78]	24.3	15.7	54.17 (9.15) [37—78]	4.9	26.8	39.07 (7.57) [26—53)	51.7	0	50 (10) [23—77]	10.6	13.3	0.13	**0.001**^c^	0.59
**SL**	48.76 (9.62) [31—69]	21.4	12.9	52.44 (8.79) [34—69]	9.8	19.5	43.55 (8.34) [31—65]	37.9	3.4	50 (10) [23—77]	12.6	12.6	0.28	0.04	0.86
**RH**	54.47 (10.49) [32—80]	5.7	25.7	50.85 (8.97) [32—72]	9.8	12.2	59.59 (10.48) [42—80]	0	44.8	50 (10) [23—77]	12.3	13.2	**0.0007**^b^	0.13	0.007
**AS**	51.97 (10.23) [33—80]	11.4	18.6	53.20 (9.60) [35—80]	4.9	19.5	50.24 (11.01) [33—72]	20.7	17.2	50 (10) [28—77]	10.2	12.7	0.11	0.69	0.15

Mean, standard deviation (in round brackets), range (in square brackets) regarding PSSI subscale t-scores as well as the percentage of pwCF and participants from the norming sample below (< 40) and above (> 60) the normal range. Note: Significant findings after a correction for multiple comparisons at q < 0.05 are printed in bold type

*PN* Paranoid, *SZ* Schizoid, *ST* Schizotypal, *BL* Borderline, *HI* Histrionic, *NA* Narcissistic, *SU* Self-insecure, *AB* Dependent, *ZW* Compulsive, *NT* Negativistic, *DP* Depressed, *SL* Selfless, *RH* Rhapsodic, *AS* Antisocial

^a^in the presence of outliers also the median is reported

^b^significant difference between pwCF and the norming sample regarding the PSSI subscales’ mean or median (PN, NA, SU)

^c^significant difference regarding the percentage of pwCF and participants from the norming sample below the norm

^d^significant difference regarding the percentage of pwCF and participants from the norming sample above the norm

Comparing the proportion of pwCF with t-values below (< 40) and above (> 60) the normal range (40–60) with the norming sample of the test based on the general population according to sex and age, pwCF had a higher proportion of subnormal t-scores regarding the subscales ‘paranoid (PN)’ (*p* = 0.001, d = 0.54; Table 2), ‘borderline (BL)’ (*p* = 2 × 10^–4^, d = 0.58), ‘self-insecure (SU)’ (*p* = 0.004, d. = 0.59), ‘negativistic (NT)’ (*p* = 2 × 10^–5^, d = 0.67), and ‘depressed (DP)’ (*p* = 0.001, d = 0.55) and a higher proportion of supranormal t-scores regarding the subscales ‘schizoid (SZ)’ (*p* = 1 × 10^–7^, d = 0.80), ‘histrionic (HI)’ (*p* = 0.003, d = 0.50), and ‘compulsive (ZW)’ (*p* = 2 × 10^–5^, d = 0.65).

When accounting for multiple testing, there was no relationship between single PSSI scale t-scores and ppFEV_1_, BMI, CFQ-R overall score, therapy adherence, and CS, despite a trend for a negative linear relationship between (1) ‘schizotypal (ST)’ t-scores and CS (*p* = 0.03, d = 0.44 [per change in 1 SD]; Table 3) and (2) ‘depressed (DP)’ t-scores and the CFQ-R overall score (*p* = 0.03, d = 0.41) as well as a positive trend for a linear relationship between (3) ‘depressed (DP)’ t-scores and CS (*p* = 0.01, d = 0.62 (per change in 1 SD)) and (4) ‘compulsive (ZW)’ t-scores and ppFEV1 (*p* = 0.04, d = 0.46).

**Table 3:** Relationship between single PSSI scales and the personality clusters in pwCF and the outcomes ppFEV1, BMI, CFQ-R, therapy adherence, and cough suppression investigated by multiple regression

	**ppFEV1**^a^				**BMI**^b^				**CFQ-R**^c^				**Adherence**^d^				**CS**^e^			
IV	b	SE	T	p	b	SE	T	p	b	SE	T	p	b	SE	T	p	b	SE	Wald χ^2^	p
**PN**	0.01	0.23	0.02	.98	0.02	0.04	0.43	.67	1.20	2.09	0.57	.57	-0.05	0.03	-1.50	.14	0.01	0.03	0.06	.81
**SZ**	-0.25	0.30	-0.82	.42	0.07	0.06	1.25	.22	-3.92	2.79	-1.41	.17	0.04	0.04	1.03	.31	0.03	0.03	0.86	.35
**ST**	0.20	0.31	0.64	.52	-0.08	0.06	-1.41	.17	-0.15	2.85	-0.05	.96	0.01	0.04	0.25	.80	-0.08	0.04	4.72	.03
**BL**	0.41	0.39	1.05	.30	0.03	0.07	0.36	.72	-0.77	3.65	-0.21	.83	-0.01	0.05	-0.26	.79	-0.06	0.04	1.62	.20
**HI**	0.61	0.34	1.80	.08	-0.04	0.07	-0.53	.60	-1.16	3.18	-0.37	.72	0.04	0.05	0.79	.43	0.05	0.04	1.42	.23
**NA**	0.26	0.32	0.82	.42	0.05	0.06	0.75	.46	-4.81	2.83	-1.70	.10	0.00	0.04	-0.08	.94	0.04	0.04	1.36	.24
**SU**	0.13	0.29	0.44	.66	0.00	0.05	0.06	.95	3.35	2.99	1.12	.27	0.07	0.04	1.96	.05	0.01	0.03	0.06	.81
**AB**	0.05	0.29	0.18	.86	0.01	0.06	0.21	.84	-3.58	2.88	-1.24	.22	0.00	0.04	-0.10	.92	0.03	0.03	0.66	.42
**ZW**	0.60	0.29	2.10	.04	0.08	0.05	1.67	.10	-3.50	2.50	-1.40	.17	-0.01	0.04	-0.25	.80	0.03	0.03	0.99	.32
**NT**	-0.01	0.40	-0.03	.98	-0.03	0.08	-0.39	.70	-3.83	3.66	-1.05	.30	0.02	0.05	0.41	.68	-0.01	0.05	0.07	.79
**DP**	-0.33	0.38	-0.87	.39	-0.03	0.07	-0.46	.65	-7.94	3.61	-2.20	.03	-0.09	0.05	-1.68	.10	0.11	0.04	6.36	.01
**SL**	0.04	0.32	0.12	.91	0.00	0.06	0.01	.99	1.07	2.81	0.38	.71	-0.03	0.04	-0.77	.45	0.00	0.03	0.00	.95
**RH**	-0.74	0.39	-1.88	.07	0.02	0.07	0.26	.79	0.91	3.56	0.26	.80	-0.02	0.05	-0.41	.68	-0.01	0.04	0.06	.81
**AS**	-0.19	0.34	-0.57	.57	-0.04	0.07	-0.57	.57	3.59	3.06	1.17	.25	0.04	0.05	0.78	.44	-0.05	0.04	1.73	.19
**Cluster**	0.96	4.89	^*^	.83	0.27	0.78	0.35	.73	-218.95	43.32	-5.05	**4 × 10**^**–6**^	-0.79	0.58	-1.36	.18	0.52	0.49	1.12	.29

*SE* Standard error, *b* Unstandardized coefficient, *T/ Wald χ*^*2*^ Test statistic, *ppFEV1* Percent predicted forced expiratory volume in one second, *CFQ-R* Cystic Fibrosis Questionnaire revised, *PN* Paranoid, *SZ* Schizoid, *ST* Schizotypal, *BL* Borderline, *HI* Histrionic, *NA* Narcissistic, *SU* Self-insecure, *AB* Dependent, *ZW* Compulsive, *NT* Negativistic, *DP* Depressed, *SL* Selfless, *RH* Rhapsodic, *AS* Antisocial. Note: Significant findings are printed in bold type

^*^bootstrapped result, 95%-confidence interval for b (-9.00; 10.82)

^a^the model is adjusted for BMI, sputum quantity, and P. aeruginosa positivity

^b^the model is adjusted for age, sex, and genotype

^c^the model is adjusted for ppFEV1

^d^the model is adjusted for sputum quantity

^e^the model is adjusted for sex

### Cluster analysis – personality traits in pwCF

**Fig. 2:**
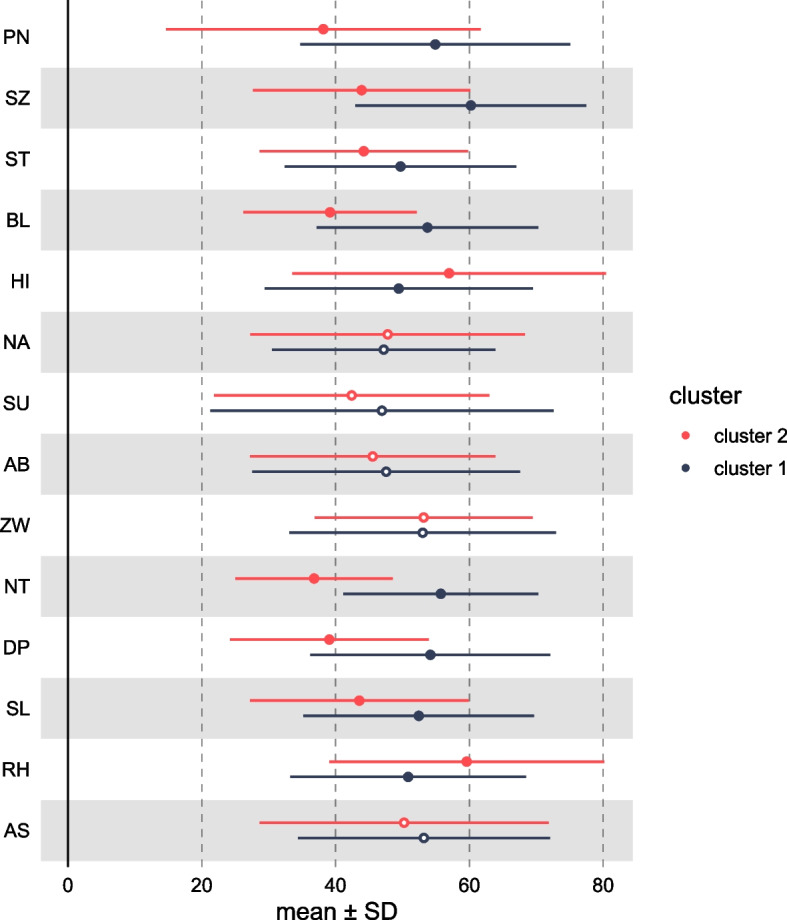
Mean PSSI Subscale Scores in pwCF for the Two Personality Clusters. The figure displays the mean ± SD. Filled dots indicate significant differences between groups at *p* < 0.05 (uncorrected). *PN* Paranoid, *SZ* Schizoid, *ST* Schizotypal, *BL* Borderline, *HI* Histrionic, *NA* Narcissistic, *SU* Self-insecure, *AB* Dependent, *ZW* Compulsive, *NT* Negativistic, *DP* Depressed, *SL* Selfless, *RH* Rhapsodic, *AS* Antisocial

Two-step cluster analysis provided fair evidence of a 2-personality cluster solution in pwCF based on PSSI subscale t-scores with a Silhouette coefficient of 0.3. The smaller cluster (cluster 2) included 29 pwCF (41.4%) and was characterized by mainly below the normal range t-scores regarding ‘negativistic (NT)’, ‘schizoid (SZ)’, ‘borderline (BL)’, ‘depressed (DP)’, and ‘paranoid (PN)’, which were the 5 most influential PSSI subscales defining the cluster structure (in descending order; Table 2, Fig. 2). The other personality cluster included pwCF, which had, on average, high normal or elevated t-scores on the same PSSI subscales outlined above. Patients from the smaller cluster were found to have a higher HRQoL, also when considering ppFEV1 as a covariate and multiple testing (*p* = 4 × 10^–6^, d = 1.32; Table 3). As revealed by exploratory subscale analysis of the CFQ-R, there was a significant difference between both clusters regarding all subscales, except for the subscales eat and digestion (Additional file 1—Table 4).

Neither of the other health outcome measures nor CS or therapy adherence was related to either personality cluster (Table 3).

## Discussion

Studies on many chronic diseases indicate an impact of personality traits on mental health and quality of life. However, in pwCF, such relationships have thus far only been reported regarding the impact of personality disorders but not personality traits, that is personality in the normative range. The purpose of the present study was, therefore, to examine the association between personality traits and HRQoL as well as the clinical parameters BMI and ppFEV_1_. Based on a standardized personality questionnaire (PSSI), accentuated personality traits were more common in 70 pwCF than in woman and men of the same age in the general population. A personality trait is considered to be accentuated if its T-value is above the normal range but does not correspond to a fully developed personality disorder. In addition, two personality clusters could be identified in pwCF based on these accentuated personality traits. The smaller of the two clusters (*N* = 29) included pwCF exhibiting t-scores at or below the normal range regarding the subscales ‘negativistic (NT)’, ‘schizoid (SZ)’, ‘borderline (BL)’, ‘depressed (DP)’, and ‘ paranoid (PD)’. The other personality cluster included pwCF (*N* = 41) which had high normal or elevated t-scores on these PSSI subscales.

### Personality in pwCF and clinical consequences

An accentuated personality, as found in the present study in pwCF, is in line with studies showing specific personality traits in other chronic diseases [6, 7, 21]. These personality traits influence the course and status as well as HRQoL in chronic conditions [6, 22]. Consistently, we found a relationship between the identified personality clusters, subsuming these accentuated personality traits, and HRQoL. PwCF with predominantly below-normal scale scores experienced a significantly higher HRQoL compared to the cluster with high normal or elevated scale values. Although low PSSI scores do not necessarily represent the ‘natural’ opposite of the trait assessed by the respective subscale and do not have to be functionally relevant [15], pwCF from this cluster are accordingly considered ‘psychologically adjusted’ while the other cluster is deemed ‘vulnerable’ in terms of HRQoL. Two implications can be derived from this. On the one hand, this result confirms the practical relevance of the purely statistically derived clusters. On the other hand, these findings point to the particular importance of non-biological determinants for HRQoL, independent of clinical outcome parameters such as ppFEV_1_ and BMI. Interestingly, there was no evidence of the functional relevance of the two personality clusters regarding clinical outcomes or adherence. This indicates that HRQoL is experienced on a subjective level that is probably not yet relevant to everyday action.

### Interplay between personality in pwCF and chronic disease

Existing studies and their findings are inconsistent regarding the causality of specific personality traits and chronic disease. There is evidence that personality is relatively stable in adulthood [23] and changes only slightly during the development of a severe illness [24]. This is based on the theoretical assumption that personality traits are shaped by our genetics [25, 26]. However, these studies address chronic diseases that occur over the lifespan, so it is questionable whether these results can be extrapolated to CF, an early-onset, congenital disease. Moreover, there have been multidimensional and systemic approaches to the diagnosis and treatment of personality traits and personality disorders since the 1980s, which are based on a biopsychosocial model [27] assuming that personality traits are sensitive to environmental influences [28]. Chronically unfavorable and often traumatic developmental conditions as experienced by pwCF since early childhood play a special role here [29, 30] and are clearly associated with a higher risk of personality disorders [31].

Interestingly, one of the two identified personality clusters in the present study is defined by pwCF with predominantly below-normal scores on five of the PSSI subscales. An explanation might be given by the concept of resilience, the psychosocial ability to cope successfully with difficult, stressful, and traumatic situations. In this way, resilience can provide quality of life and well-being despite chronic illness [32]. Based on this, we assume that pwCF of the psychologically adjusted personality cluster could have developed a high level of resilience in dealing with stressors related to their illness. This is consistent with the observation that at least a subpopulation of pwCF shows a high degree of resilience [33]. Unfortunately, no such information is available in the current study.

### Implications

In the medical setting, patients with accentuated personality traits or personality disorders often represent a challenge to the medical team because of their problematic interaction style. For example, they formulate their needs inadequately, have unrealistic expectations of their treatment (e.g., regarding time resources), and are often less reliable and adherent [34, 35]. The better the medical team responds to the patient's personality, the more successful it will be in building a positive and sustainable relationship that enables the patient to formulate his needs adequately and to feel accepted [36]. By psychological counseling, the nature and severity of the impact of personality traits on oneself and others and the costs of having a conflictual interaction style can be outlined. Moreover, alternative behavior patterns can be discussed and practiced. Thus, a process of change might be initiated that helps the patient obtain more confidence in medical treatment, increase adherence and thereby improve HRQoL. For this reason, specific diagnostics are important for identifying pwCF with accentuated personality traits.

### Limitations

A weakness of the present study is the sample size. Although the sample size was relatively large for a single-center CF study, it may not have been large enough to detect the complexities in the relationship between personality and HRQoL and physiological outcomes. For example, there was a trend for a significant difference between the PSSI t-scores of pwCF and a scale mean of 50 regarding `schizoid´ and `compulsive´. Additionally, the study does not provide information on the factors involved in developing accentuated personality traits in pwCF across the lifespan. Longitudinal studies are needed that follow pwCF across their lifespan and determine variables such as life events, relationships with parents and peers, and protective factors.

Likewise, factors that additionally may influence HRQoL were not recorded. Thus, it is conceivable that the presence of mental disorders can also explain the reduced HRQoL.

### Conclusions

In this first study to address personality traits in pwCF, we found that pwCF showed a higher prevalence of certain personality traits related to personality disorders than the norming sample. Moreover, we found two personality clusters within the sample of pwCF that have consequences for psychological well-being. Thus, our study has three main implications. First, in pwCF, HRQoL is mainly determined by psychological factors, including personality. Second, since more recent personality theories assume that personality is modifiable, our findings imply that patients with a specific set of personality traits may benefit from psychological support. Third, for more targeted psychological support, future studies need to (prospectively) address risk and protective factors contributing to developing certain personality traits in pwCF.

## Supplementary Information

**Additional file 1.**

## Data Availability

The datasets used and/or analyzed during the current study are available from the corresponding author upon reasonable request.

## Abbreviations

CS: Cough suppression
CF: Cystic fibrosis
HRQoL: Health-related quality of life
ppFEV_1_: Percent predicted forced expiratory volume in one second
BMI: Body mass index
CFRD: CF-related diabetes
pwCF: People with cystic fibrosis
CFQ-R: Cystic Fibrosis Questionnaire for adolescents and adults over 14 years old
CFTR gene: Cystic fibrosis transmembrane conductance regulator gene
PSSI: Persönlichkeits-Stil- und Störungs-Inventar
DSM-IV: Diagnostic and Statistical Manual of Mental Disorders 4^th^ edition
ICD-10: International Classification of Diseases 10^th^ Revision
