# Supplementary material for: How personality influences health outcomes and quality of life in adult patients with cystic fibrosis
Source: BMC Pulm Med. 2023 Jun 1;23:190. doi: 10.1186/s12890-023-02463-y (PMC10233905; doi:10.1186/s12890-023-02463-y)
Supplement: Supplementary file 1 — Additional file 1. [file 12890_2023_2463_MOESM1_ESM.docx]

**Additional Material**

**How Personality Influences Health Outcomes and Quality of Life in Adult Patients with Cystic Fibrosis**

Ute Niehammer^a^, Svenja Straßburg^a^ MD, Sivagurunathan Sutharsan^a^ MD, Christian Taube^a^ MD, Matthias Welsner^a^ MD, Florian Stehling MD, Raphael Hirtz MD PhD

**Overview**

1. Additional Methods – Covariate Selection and Testing of the Statistical Assumptions
2. Additional Table 1 **-** PSSI: recorded personality traits and disorders of personality
3. Additional Table 2 – Question and response choices of the self-report questionnaire
4. Additional Table 3 – Demographic und clinical characteristics regarding patients with information on the CFQ-R
5. Additional Table 4 – Relationship between Personality Clusters and CFQ-R subscales
6. Additional Table 5 – Bivariate Correlations between PSSI Subscales
7. Additional References

**Additional** **Methods**

**Coding of Variables**

Categories of CS were collapsed into 3 categories (category 1: ‘never’ and ‘seldom’; category 2: ‘occasionally’; category 3: ‘frequently’ and ‘always’), and the 4 categories of sputum quantity were reclassified into 2 categories (category 1: ‘no’ and ‘little’; category 2: ‘moderate’ and ‘substantial’). The variable therapy adherence was coded by summing scores on the items self-administered physical therapy (none = 0, ≤ 1x/week = 1, 2-3x/week= 2; every day = 3), supervised physical therapy (none = 0, < 1x/week = 1, 1x/week = 2, > 1x/week = 3), and exercise (none = 0, < 1x/week = 1, 1x/week = 2, > 1x/week = 3).

**Additional** **Selection**

Considering the sample size of the present study and the large number of potential covariates for regression analysis (age, gender, genotype, cough frequency, sputum quantity, diabetes, exocrine pancreatic insufficiency, Pseudomonas colonization status, employment status, marital status), an analysis-specific subset of covariates with a significant correlation with either outcome measures (ppFEV1, BMI, CFQ-R overall and subscale scores) as well as cough suppression and therapy adherence (1) was chosen. Correlation analyses were performed considering the scale of measure and the presence of outliers. This step of analysis was considered exploratory.

**Multiple Regression – Testing of Assumptions**

Normality of the residuals of the multiple regression analyses regarding the outcome measures FEV1, BMI, and CFQ-R (overall and subscale) scores as well as cough suppression and therapy adherence (dependent variables) were assessed by the Kolmogorov-Smirnov test. Outlier detection relied on standardized residuals exceeding ­± 3 SD. Multicollinearity was defined by variance inflation factors exceeding 10 and homoscedasticity was evaluated by the Breusch-Pagan test. Autocorrelations of residuals were excluded by the Durbin-Watson test. Linearity was checked by visual inspection of (partial) bivariate scatter plots between the DV of interest and all IVs as well as all pairs of IVs.

**Logistic Regression – Testing of Assumptions**

Linearity of the relationship between continuous predictors and the logit transform of perspectives was assessed by the Box-Tidwell approach. As a goodness-of-fit measure relying on a χ2 statistic was employed, all pairs of categorial variables were inspected for sufficient cell size. The parallel lines test verified equivalent slope coefficients across the response categories of perspectives.

**Cluster analysis –Assumptions, Outlier Detection, and Cluster Structure**

Cluster analysis assumes that the variables subjected to analysis are independent and normally distributed. Independence of the variables subjected to cluster analysis was evaluated by correlation analyses considering the presence of outliers and normality. The latter was tested by the Kolmogorov-Smirnov test. As can be seen from Supplementary Table 5, several PSSI subscales were correlated, and not all subscales were normally distributed (`schizoid (SZ)´, `schizotypal (ST)´ `compulsive (ZW)´). However, two-step cluster analysis is robust to a violation of these assumptions (2).

Outliers were accounted for by standard noise handling at a 25% threshold, but considering the relatively small sample size, the number of maximum branches per leaf node was set to 4 for more sensitive outlier detection.

To assess the evidence of a cluster structure regarding the PSSI subscales, we determined the Silhouette measure as implemented in SPSS.

**ANCOVA Analysis – Testing of Assumptions**

Normality of studentized residuals regarding the respective dependent variabel (ppFEV1, BMI, CFQ-R overall score, therapy adherence) was assessed by the Kolmogorov-Smirnov test. In case of non-normally distributed residuals or the presence of outliers, the dependent variable as rank-transformed according to Templeton (3), preserving its mean and standard deviation. If normality was not achieved by this approach, bootstrapping with 1000 bootstrap samples was performed. Equality of variances was tested by Levene's test. In the presence of heteroscedasticity, a robust standard error estimator for regression coefficients according to Davidson-McKinnan (HC3) was applied. Homogeneity of regression was investigated by separate ANCOVAs testing for the significance of interactions between the independent variable (personality clusters) and the considered covariates (1).

**Additional** **Table 1 -** PSSI: recorded personality traits and disorders of personality

| Skala | Personality trait | Personality disorder | Item as an example |
| --- | --- | --- | --- |
| PN | idiosyncratic | paranoid | Sometimes I don`t trust my friends either. |
| SZ | reserved | schizoid | Closeness to other people makes me rather uncomfortable. |
| ST | foreboding | schizotypal | I believe in thought transmission. |
| BL | spontaneous | borderline | My feelings often change abruptly and impulsively. |
| HI | kindly | histrionic | I can be very charming. |
| NA | ambitious | narcissistic | I have many dreams and ideals. |
| SU | self-critical | self-insecure | Speaking in front of many people is difficult for me. |
| AB | loyal | dependent | When I'm all alone, I often feel helpless. |
| ZW | careful | compulsive | Accuracy and order are very important to me. |
| NT | critical | negativistic | I have often been treated unfairly in my life. |
| DP | passive | depressed | I often have feelings of guilt. |
| SL | helpful | selfless | When others need me, I am always ready to help. |
| RH | optimistic | rhapsodic | Wherever I go, I spread good cheer. |
| AS | Self-assertive | antisocial | I don't take shit from others. |

**Additional** **Table 2** - Question and Response Choices of the Self-Report Questionnaire to Gain Information About CS, Therapy Adherence, Diagnosis Disclosure

| **Question** | **Response choices** |
| --- | --- |
| How do you assess your health perspectives in future? | Excellent \| Moderate \| Bad |
| How do you assess your frequency of coughing in clinically stable condition? | I'm coughing…  Not at all \| Little \| Moderate \| A lot |
| Is there mucus when you are in clinically stable condition? | No \| Yes, a little (one tablespoon) \| Yes, moderate (one eggcup) \| Yes, a lot (one cup) |
| When you are in public: Do you suppress coughing? | Never \| Seldom \| Occasionally \| Frequently \| Always |
| How frequent do you visit an assisted physical therapy? | Never \| Less than 1x/week \| 1x/week \| More than 1x/week |
| How frequent do you practice self-administered physical therapy? | Never \| 1x/day \| 2-3x/week \| 1x/week \| Less than 1x\|week |
| How frequent do you take your inhaled medication? | Never \| 2-3x/day \| 1x/day \| Several times a week \| Less than 1x\|week |
| Do you exercise? | Yes \| No |
| If so: How often do you exercise? | Less than 1x/week \| 1x\|week \| More than 1x/week |
| Does your family know about your disease? | Yes \| No \| Partly |
| Do your friends know about your disease? | Yes \| No \| Partly |
| Does your employer know about your disease? | Yes \| No \| Partly |
| Does your colleagues know about your disease? | Yes \| No \| Partly |

**Additional** **Table 3** - Demographic and Clinical Characteristics Regarding Patients with Information on the CFQ-R

|  | All subjects (n=70) | Information on the  CFQ-R (n=63) | No information on the  CFQ-R (n=7) |
| --- | --- | --- | --- |
| Age, years | 32.71 ± 11.65  (18-71) | 32.98 ± 12.06  (18-71) | 30.29 ± 6.95  (24-43) |
| Sex (female) n, % | 29 (41) | 26 (41) | 3 (43) female |
| Genotype  n, (%)  *F508del homozygous*  *F508del heterozygous*  *Other* | 28 (40)  31 (44)  11 (16) | 24 (38)  30 (48)  9 (14) | 28 (40)  31 (44)  11 (16) |
| BMI, kg/m^2^ | 20.32 ± 3.41  (15-33) | 20.41 ± 3.45  (15-33) | 19.51 ± 3.14  (16-25) |
| ppFEV_1_ | 43.27 ± 19.93  (16-99) | 44.56 ± 20,24  (16-99) | 31.71 ± 12.71  (16-50) |
| Pancreatic insufficiency  n, % | 45 (64) | 40 (63.5) | 5 (71) |
| CF-related diabetes  n, % | 22 (31) | 20 (32) | 2 (29) |
| P. aeruginosa positive  n, % | 52 (74) | 46 (73) | 6 (86) |
| Hospital  n, %  *inpatient*  *outpatient* | 67 (96)  3 (4) | 60 (95)  3 (5) | 7 (100)  0 |
| Reasons for medical treatment  n, %  *PEX*  *Starting CFTR-*  *Modulator Therapy*  *IVAT*  *Others* | 33 (47)  14 (20)  8 (11)  16 (22) | 28 (44)  13 (21)  7 (11)  15 (24) | 33 (47)  14 (20)  8 (11)  16 (22) |
| Marital status  n,%  *single*  *in partnership / married* | 36 (51)  34 (49) | 32 (51)  28 (49) | 4 (57)  3 (43) |

Results are presented as mean ± and standard deviation (SD) and range or number of patients n (%), ppFEV_1_ - percent predicted forced expiratory volume in one second, PEX - pulmonary exacerbation; IVAT – intravenous antibiotic therapy (prophylactic). There was no significant difference between subjects with and without information on the CFQ-R as investigated by two-sample t-tests and χ2-tests of independence.

| **Additional** **Table 4** - Relationship between Personality Clusters and CFQ-R Subscales | | | | | | |
| --- | --- | --- | --- | --- | --- | --- |
|  |  |  | 95%-CI for b | |  |  |
|  | b | SE | lower CI | upper CI | T | p |
| **physical** | -14.91 | 5.42 | -25.72 | -4.10 | -2.75 | .008 |
| **vitality** | -17.17 | 4.90 | -26.95 | -7.40 | -3.51 | 8x10^-4^ |
| **emotion** | -25.10 | 4.30 | -33.69 | -16.51 | -5.83 | 2x10^-7^ |
| **eat** | -11.17 | 6.67 | -23.23 | 1.32 | - | .09 |
| **treatment burden** | -11.22 | 4.98 | -21.17 | -1.28 | -2.25 | .03 |
| **health perception** | -22.63 | 4.82 | -32.25 | -13.01 | -4.70 | 1x10^-5^ |
| **social** | -19.33 | 4.34 | -28.00 | -10.66 | -4.45 | 3x10^-5^ |
| **body** | -27.81 | 5.87 | -39.53 | -16.09 | -4.74 | 1x10^-5^ |
| **role** | -18.90 | 6.49 | -31.87 | -5.92 | -2.91 | .005 |
| **weight** | -17.63 | 7.37 | -32.34 | -2.92 | -2.39 | .02 |
| **respiratory** | -9.90 | 4.81 | -19.50 | -0.29 | -2.06 | .04 |
| **digestion** | -11.49 | 6.84 | -23.90 | 0.09 | - | .11 |

b = unstandardized coefficient including the 95%-confidence interval (CI), SE = standard error, T = test statistic. The CFQ-R subscales emotion, weight, and digestion were transformed as detailed in the methods section. The results regarding the CFQ-R subscales eat and digestion were bootstrapped. Please note: a negative algebraic sign regarding the unstandardized regression coefficient implies higher values in pwCF from cluster 2. All analyses were adjusted for ppFEV1.

| **Additional** **Table 5** - Bivariate Correlations between PSSI Subscales | | | | | | | | | | | | | | |
| --- | --- | --- | --- | --- | --- | --- | --- | --- | --- | --- | --- | --- | --- | --- |
|  | PN | SZ | ST | BL | HI | NA | SU | AB | ZW | NT | DP | SL | RH | AS |
| PN | 1.00 |  |  |  |  |  |  |  |  |  |  |  |  |  |
| SZ | 0.44^**^ | 1.00 |  |  |  |  |  |  |  |  |  |  |  |  |
| ST | 0.15 | 0.19 | 1.00 |  |  |  |  |  |  |  |  |  |  |  |
| BL | 0.28^**^ | 0.42^**^ | 0.39^**^ | 1.00 |  |  |  |  |  |  |  |  |  |  |
| HI | -0.09 | -0.35^**^ | 0.13 | -0.25^*^ | 1.00 |  |  |  |  |  |  |  |  |  |
| NA | 0.01 | -0.18^*^ | 0.28^**^ | -0.01 | 0.28^**^ | 1.00 |  |  |  |  |  |  |  |  |
| SU | 0.02 | 0.02 | 0.05 | 0.30^**^ | -0.35^**^ | -0.05 | 1.00 |  |  |  |  |  |  |  |
| AB | -0.03 | -0.14 | 0.29^*^ | 0.23 | -0.12 | 0.16 | 0.32^**^ | 1.00 |  |  |  |  |  |  |
| ZW | 0.08 | 0.03 | -0.07 | -0.10 | 0.13 | 0.02 | -0.08 | 0.03 | 1.00 |  |  |  |  |  |
| NT | 0.54^**^ | 0.69^**^ | 0.31^**^ | 0.65^**^ | -0.22 | -0.01 | 0.09 | 0.04 | 0.09 | 1.00 |  |  |  |  |
| DP | 0.29^**^ | 0.50^**^ | 0.32^**^ | 0.77^**^ | -0.42^**^ | -0.04 | 0.41^**^ | 0.22 | -0.21 | 0.62^**^ | 1.00 |  |  |  |
| SL | 0.17^*^ | 0.34^**^ | 0.26^*^ | 0.43^**^ | -0.04 | 0.05 | 0.16 | 0.17 | 0.18 | 0.47^**^ | 0.31^**^ | 1.00 |  |  |
| RH | -0.12 | -0.33^**^ | 0.07 | -0.40^**^ | 0.68^**^ | 0.21* | -0.18^*^ | 0.15 | 0.35^**^ | -0.26^*^ | -0.51^**^ | 0.14 | 1.00 |  |
| AS | 0.22^**^ | 0.16 | 0.03 | -0.17 | 0.34^**^ | 0.24^**^ | -0.34^**^ | -0.07 | 0.20 | 0.25^*^ | -0.23 | -0.08 | 0.29^*^ | 1.00 |

PN = paranoid, SZ = schizoid, ST = schizotypal, BL = borderline, HI = histrionic, NA = narcissistic. SU = self-insecure, AB = dependent, ZW = compulsive, NT = negativistic, DP= depressed, SL= selfless, RH = rhapsodic, AS = antisocial. Regarding the PSSI subscales PN, NA, and SU, Kendall's  is reported considering all pairs of bivariate correlations, otherwise Pearson correlations are reported. ^*^ *p* < .05. ^**^ *p* < .01

**Additional References**

1. Tabachnick BG, Fidell LS, Ullman JB. Using multivariate statistics: Pearson Boston, MA; 2007.

2. Janssen J, Laatz W. Statistische Datenanalyse mit SPSS: eine anwendungsorientierte Einführung in das Basissystem und das Modul Exakte Tests: Springer-Verlag; 2016.

3. Templeton GF. A two-step approach for transforming continuous variables to normal: implications and recommendations for IS research. Commun Assoc Inf Syst. 2011;28(1):4.
